# Supplementary material for: Protein–ligand binding with the coarse-grained Martini model
Source: Nat Commun. 2020 Jul 24;11:3714. doi: 10.1038/s41467-020-17437-5 (PMC7382508; doi:10.1038/s41467-020-17437-5)
Supplement: Supplementary file 1 — Supplementary Information [file 41467_2020_17437_MOESM1_ESM.pdf]

Supplementary Information for:

Protein-ligand binding with the coarse-grained Martini model

Paulo C. T. Souza[1], Sebastian Thallmair[1], Paolo Conflitti[2], Carlos Ramírez Palacios[1], Riccardo Alessandri[1], Stefano Raniolo[2], Vittorio Limongelli[2,3]*, Siewert J. Marrink[1]**

[1]Groningen Biomolecular Sciences and Biotechnology Institute and Zernike Institute for Advanced Materials, University of Groningen, Nijenborgh 7, 9747 AG Groningen, Netherlands

[2]Faculty of Biomedical Sciences, Institute of Computational Science, Università della Svizzera italiana (USI), via G. Buffi 13, CH-6900 Lugano, Switzerland

[3]Department of Pharmacy, University of Naples "Federico II", via D. Montesano 49, I-80131 Naples, Italy.

* Correspondence and requests for materials should be addressed to P.C.T.S. (e-mail: paulocts @gmail.com), to V.L. (e-mail: vittoriolimongelli@gmail.com), or to S.J.M. (e-mail: s.j.marrink@rug.nl).

These authors contributed equally: Paulo C. T. Souza , Sebastian Thallmair

TABLE OF CONTENT:

	Page
S1. SUPPLEMENTARY FIGURES	*S3*
S2. SUPPLEMENTARY TABLES	*S11*
S3. SUPPLEMENTARY METHODS	*S19*
S4. SUPPLEMENTARY DISCUSSION	*S31*
S5. REFERENCES	*S41*

S1. SUPPLEMENTARY FIGURES

In the following pages the Supplementary Figures 1–8 are depicted.

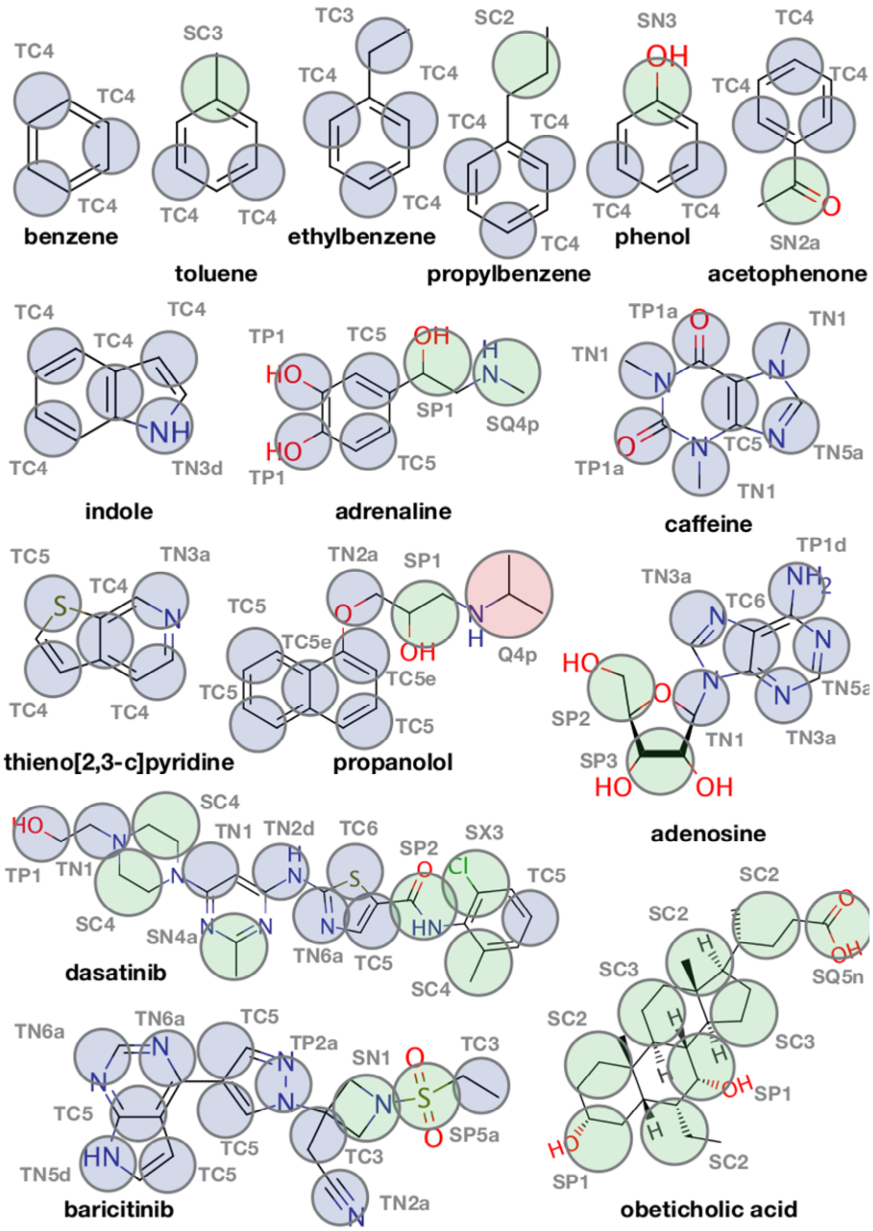

Supplementary Figure 1. CG mappings of the small-molecules used in this work. CG beads are shown over the atomistic structures of the ligands. The (Martini) sizes of the beads are represented with different colors: regular, small, and tiny beads are represented in red, green, and blue, respectively.

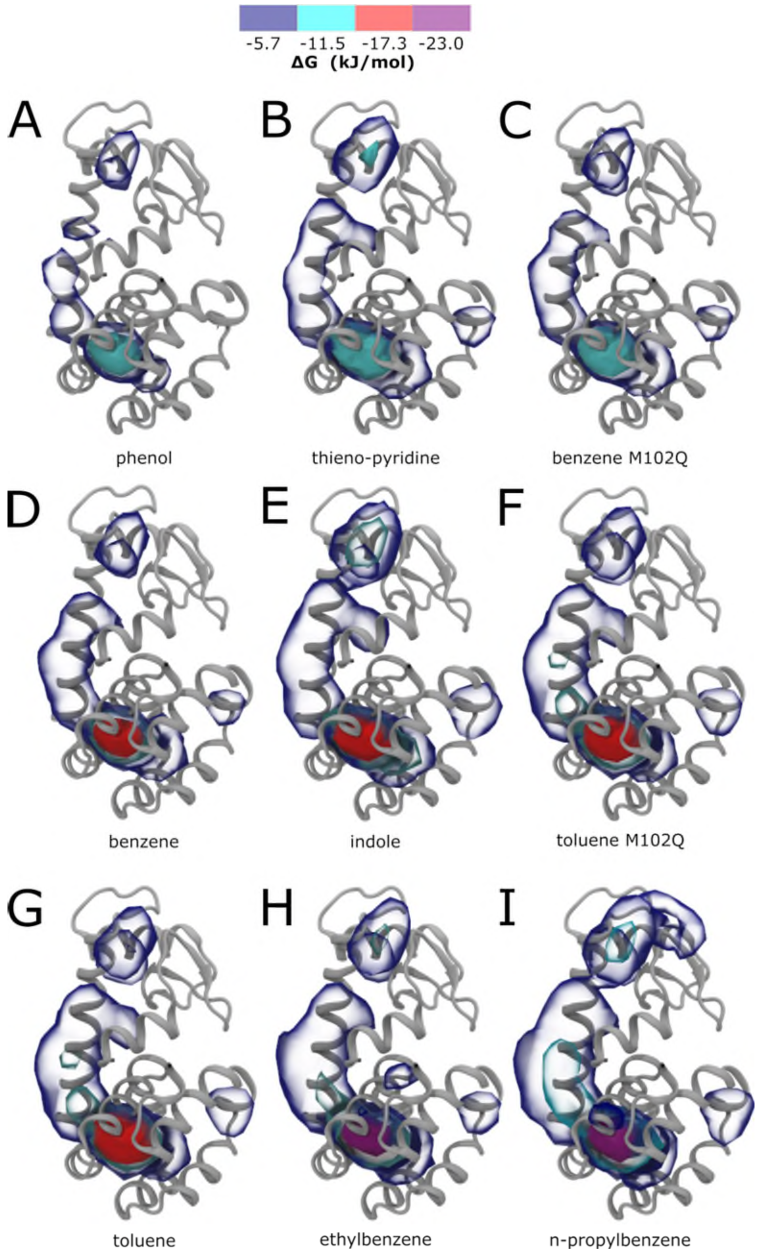

Supplementary Figure 2. Ligand densities around L99A single and L99A/M102Q double mutants of T4 lysozyme obtained from averaging 0.9 ms of CG simulations. The blue, cyan, red, and violet isosurfaces correspond to a 10, 100, 1,000, and 10,000 fold higher ligand density than in water. These densities translate to the free energy values shown at the color map. The ligands are (A) phenol, (B) thieno-pyridine, (C) benzene (L99A/M102Q), (D) benzene, (E) indole, (F) toluene (L99A/M102A), (G) toluene, (H) ethylbenzene, and (I) n-propylbenzene.

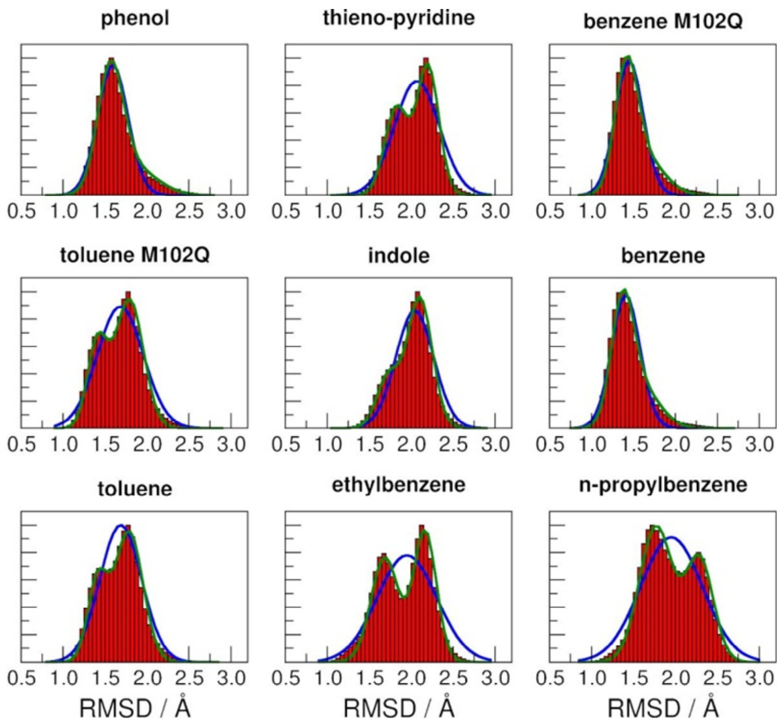

Supplementary Figure 3. Histograms of the RMSD of the ligands phenol, thieno-pyridine, benzene, toluene, indole, ethylbenzene, and n-propylbenzene together with the contact protein beads. The blue line depicts the fit using a single Gaussian; the green line is fitted using two Gaussian functions. The corresponding fitting parameters are given in Table 1 of the manuscript and Supplementary Table 1.

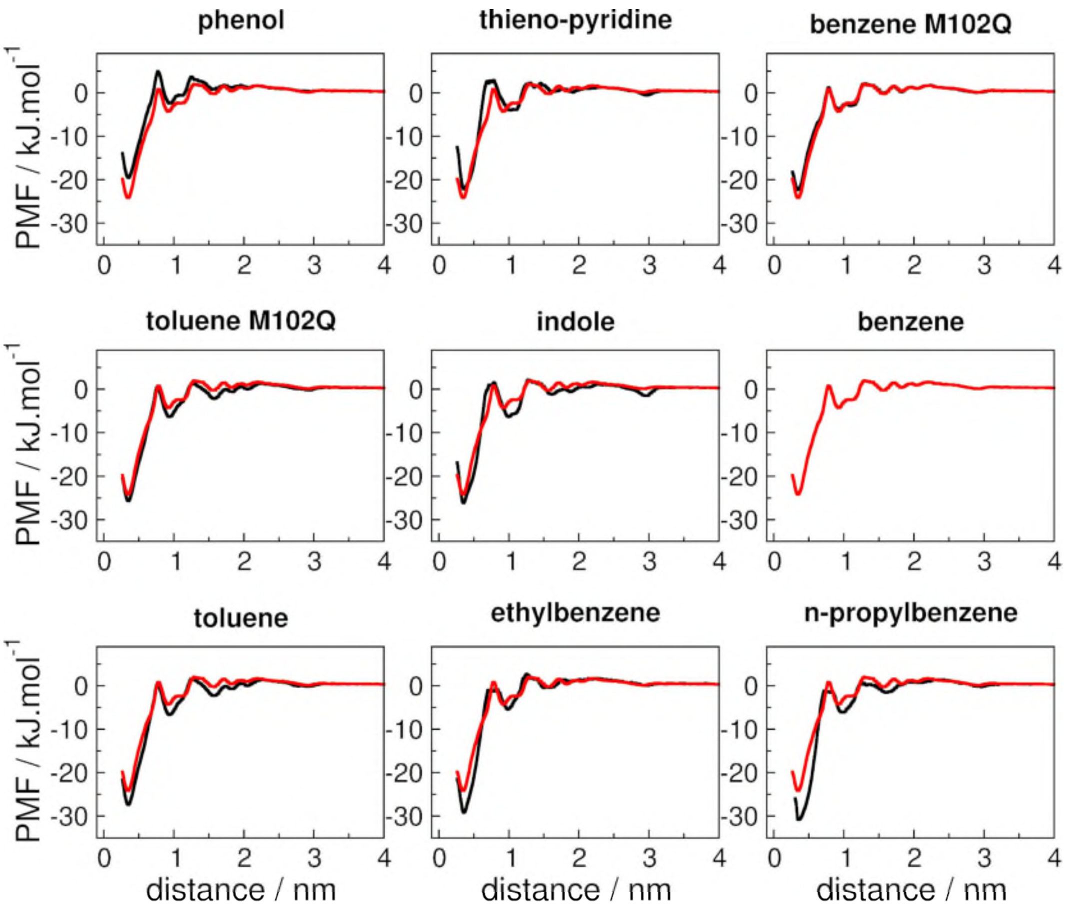

Supplementary Figure 4. Radial ligand-receptor potentials of mean-force (PMFs) obtained from unbiased MD simulations with the respective ligands (black line). The red line depicts the PMF of benzene for comparison.

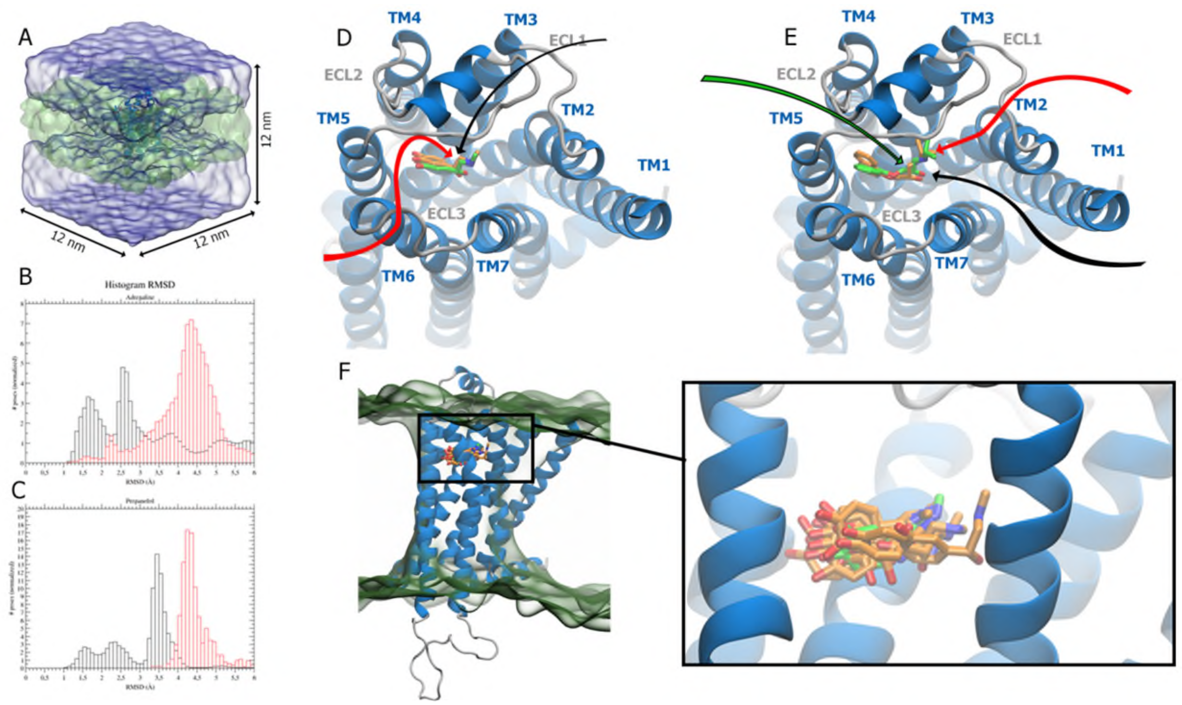

Supplementary Figure 5. Coarse-grained simulations of adrenaline / propanolol binding to β2AR. A) Simulation box containing β2AR embedded in a POPC bilayer (green) and 10 adrenaline molecules (red) solvated in water (transparent blue surface). B/C) Histograms of RMSD of all the binding poses of adrenaline (B) and propanolol (C) observed during the pre-binding (red bars) and the binding simulations (black bars). D/E) Comparison of the crystallographic binding mode of adrenaline (D) and propanolol (E) (green) and the best binding poses obtained from the binding simulations (orange). The protein is depicted in cartoon, with random coils and turns colored in silver and alpha helices in blue. The molecules are shown in licorice with nitrogen atoms in blue, oxygens in red and carbon atoms in green or orange. The most recurrent binding pathways are shown as black, red and green solid lines. F) Multiple binding poses of adrenaline obtained from the CG model by back-mapping. Only structures with an RMSD below 2.1 Å with respect to the crystallographic ligand binding mode are shown.

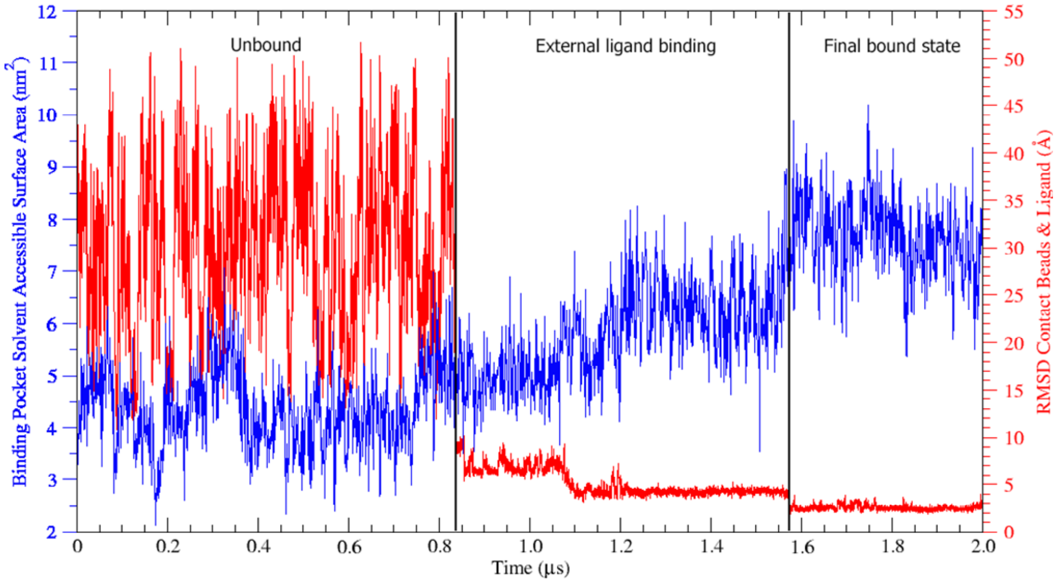

Supplementary Figure 6. Evolution of the RMSD of the contact beads and ligand (red solid line) and the Solvent Accessible Surface Area (SASA) of the binding pocket (blue solid line) during binding of obeticholic acid to FXR. The plot is divided in three regions: the unbound, the external ligand binding and the final bound state conformations for the sake of clarity.

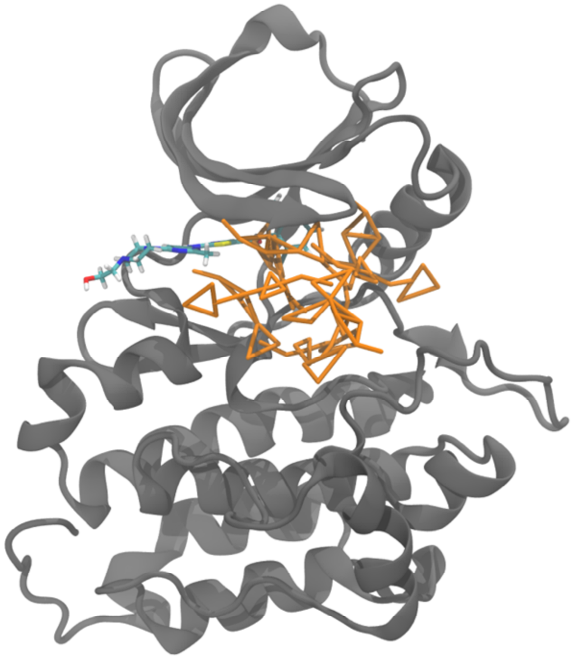

Supplementary Figure 7. Agglomerate formed when adding five molecules of dasatinib (orange) per simulation box. The dasatinib molecules do not aggregate in the water phase, but after 500 ns of simulation they start to accumulate in a pre-binding region located between the two protein lobules. During most of the remainder of the trajectory dasatinib stays in the agglomerate, thereby reducing the effective concentration in the solvent. Binding rate was therefore lower when compared to simulations with a single ligand molecule per box. Only one binding event was observed in the 40 μs trajectory.

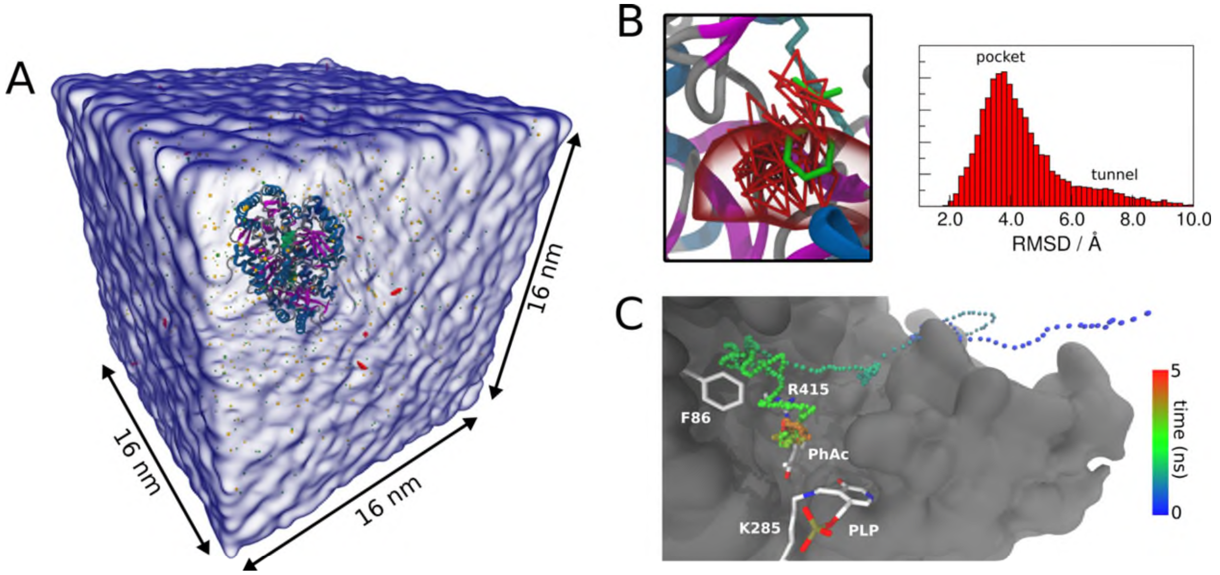

Supplementary Figure 8. Unbiased simulations of acetophenone binding to *Vf*-ATA at the CG Martini level. A) Simulation box containing the dimeric *Vf*-ATA and ten acetophenone molecules (red) solvated in water (transparent blue surface). B) DFT-optimized structure of acetophenone (green) in the binding pocket aligned to the *Vf*-ATA structure. In addition, several CG snapshots of acetophenone (red) and the acetophenone density in the binding pocket (transparent red isosurface) are shown. The RMSD histogram of acetophenone and the contact protein beads in relation to the DFT optimized structure is depicted on the right side. C) Binding event of acetophenone to the active site of *Vf*-ATA. The colored spheres indicate the position of the center of mass of acetophenone. Before arriving to the active site, acetophenone passes by the residues Arg415 – also known as switching arginine – and Phe86. The surface of the protein is shown as a gray surface. The PLP cofactor bound to Lys285 marks the end of the ~15 Å deep tunnel towards the active site. The atomistic substrate position corresponds to a superimposition of the coordinates of a DFT-optimized binding site.[1]

S2. SUPPLEMENTARY TABLES

In the following pages the Supplementary Tables 1–9 are given.

Supplementary Table 1. RMSD of the ligand binding to T4 lysozyme fitted using two Gaussian functions and their relative contribution to the total binding.

Ligands[a)]	non-binders			binders					
	phenol	thieno-pyridine	benzene M102Q	benzene	indole	toluene M102Q	toluene	ethyl-benzene	n-propyl-benzene
RMSD [Å]	1.6±0.1	1.8±0.1	1.4±0.1	1.4±0.1	1.7±0.1	1.4±0.1	1.4±0.1	1.7±0.2	1.8±0.2
rel. population	0.82	0.40	0.78	0.75	0.28	0.40	0.38	0.44	0.57
RMSD [Å]	1.8±0.3	2.2±0.1	1.6±0.2	1.6±0.2	2.1±0.1	1.8±0.2	1.8±0.2	2.2±0.1	2.3±0.2
rel. population	0.18	0.60	22.0	0.25	0.72	0.60	0.62	0.66	0.43
R^2	0.99	0.99	0.99	0.99	0.99	0.99	0.99	0.99	0.99
reference pdb code	1LI2	185L	181L	181L	185L	4I7K	4W53	4W54	4W55

[a)] Ligand names containing “M102Q” indicate the systems simulated with the L99A/M102Q double mutant of T4 lysozyme. The rest of the MD simulations were performed with the single mutant L99A.

Supplementary Table 2. Benchmark tests performed with Martini 3 and CHARMM.

# CPU's	CHARMM all-atom (AA)			Martini 3 coarse-grained (CG)			Performance ratio: AA/CG
	# particles	performance (ns/day)		# particles	performance (ns/day)		
		per replica	average		per replica	average	
4	44,425	149.0<br>141.1<br>145.2	144.8±3.5	3,928	16,318.1<br>16,343.3<br>16,491.3	16,384.2±94.6	113.18
12	355,400	16.8<br>16.4<br>17.1	16.8±0.3	31,424	4,715.8<br>4,697.6<br>4,632.1	4,681.8±44.0	278.96
12	710,800	7.9<br>6.8<br>8.4	7.7±0.8	62,848	2,722.5<br>2,711.7<br>2,698.9	2,711.0±11.8	351.50

Supplementary Table 3. Diffusion coefficients (10^{-5} cm^2/s): comparison of experiments and Martini 3 CG simulations.

Molecule	Experiment	CG simulation	CG/Exp
ethylbenzene	0.89	2.40 ± 0.38	2.7
cyclohexane	0.89	2.40 ± 0.05	2.7
toluene	0.93	2.89 ± 0.24	3.1
phenol	0.10	3.03 ± 0.32	3.0
benzene	0.11	3.18 ± 0.40	2.9

Supplementary Table 4. Simulation parameters for all CG simulations of protein systems discussed in the present work (for abbreviations see footnote [a]).

	T4L	$A_{2A}R$	β2AR	FXR	c-Src	*Vf*-ATA	AAK1
cutoff scheme	Verlet	Verlet	Verlet	Verlet	Verlet	Verlet	Verlet
buffer tolerance [kJ mol^{-1}]	0.005	0.005	0.005	0.005	0.005	0.005	0.005
Coulomb scheme	RF	RF	RF	RF	RF	RF	RF
cutoff [nm]	1.1	1.1	1.1	1.1	1.1	1.1	1.1
ε_r	15	15	15	15	15	15	15
van-der-Waals scheme	cutoff	cutoff	cutoff	cutoff	cutoff	cutoff	cutoff
cutoff [nm]	1.1	1.1	1.1	1.1	1.1	1.1	1.1
thermostat	VR	VR	VR	VR	VR	VR	VR
τ_T [ps]	1.0	1.0	1.0	1.0	1.0	1.0	1.0
reference T [K]	310	320	310	310	310	310	310
barostat	PR	PR	PR	PR	PR	PR	PR
τ_p [ps]	12.0	12.0	12.0	12.0	12.0	12.0	12.0
reference p [bar]	1.0	1.0	1.0	1.0	1.0	1.0	1.0
minimization algorithm	SD	SD	SD	SD	SD	SD	SD
number of steps	500	$5 \cdot 10^6$	$5 \cdot 10^6$	$5 \cdot 10^6$	500	500	500
equilibration integrator	LF	LF	LF	LF	LF	LF	LF
Δt [ps]	0.01	0.01	0.01	0.01	0.01	0.01	0.01
number of steps	$1.0 \cdot 10^5$	$1.0 \cdot 10^6$	$1.0 \cdot 10^6$	$1.0 \cdot 10^6$	$5.0 \cdot 10^4$	$5.0 \cdot 10^4$	$1.0 \cdot 10^5$
total time [ns]	1.0	10	10	10	0.5	0.5	1.0
production integrator	LF	LF	LF	LF	LF	LF	LF
Δt [ps]	0.02	0.02	0.02	0.02	0.02	0.02	0.02
number of steps	$1.5 \cdot 10^9$	$1.0 \cdot 10^9$	$1.0 \cdot 10^9$	$1.0 \cdot 10^9$	$1.5 \cdot 10^9$	$1.0 \cdot 10^9$	$1.5 \cdot 10^9$
total time [µs]	30	20	20	20	30	15	30
number of simulations	9 × 30	2 × 12	2 × 12	72	10	10	30

[a] abbreviations: T4L = T4 lysozyme; LF = leap frog; PR = Parrinello-Rahman; RF = reaction field; SD = steepest decent; VR = velocity rescale;

Supplementary Table 5. Additional simulation settings for the ligand model validation discussed in Supplementary Methods.

	T4 lysozyme	$A_{2A}R$	β2AR / FXR	*Vf*-ATA
ligands	benzene, phenol, indole, n-propylbenzene	caffeine, ribose, adenine	adrenaline, propranolol / obeticholic acid	acetophenone
free energy method	thermodynamic integration	thermodynamic integration	thermodynamic integration	thermodynamic integration
integrator	stochastic dynamics	stochastic dynamics	stochastic dynamics	stochastic dynamics
number of λ points	21	21	40	20
Δt [ps]	0.02	0.02	0.02	0.02
number of steps / λ point	$2.0 \cdot 10^5$	$2.0 \cdot 10^5$	$4.0 \cdot 10^5$	$4.0 \cdot 10^5$
soft core potential	yes	yes	yes	yes
α	0.5	0.5	0.5	0.5
σ	0.3	0.3	0.3	0.3
power	1	1	1	1
r-power	6	6	6	6

Supplementary Table 6. System setup for all CG simulations of protein systems discussed in the present work.

	T4 lysozyme	$A_{2A}R$	β2AR	FXR	c-Src	*Vf*-ATA	AAK1
pdb structure	181L	3RFM	6MXT	1OSV	1Y57	4E3Q	5L4Q
box size (x×y×z) [nm^3]	10 × 10 × 10	12 × 12 × 12	12 × 12 × 12	10 × 10 × 10	12 × 12 × 12	16 × 16 × 16	10 × 10 × 10
membrane composition	—	POPC	POPC	—	—	—	—
number of CG water	8,846	~8,800	~8,900	8,368	14,532	33,268	8,642
NaCl conc. [M]	0.14	0.15	0.15	0.15	0.14	0.15	0.15
number of ligands	1	7 / 10 / 13	10	4	1	10	1
ligand conc. [M]	$1.6 \cdot 10^{-3}$	$11 \cdot 10^{-3}$/ $16 \cdot 10^{-3}$/ $20 \cdot 10^{-3}$	$16 \cdot 10^{-3}$	$6.6 \cdot 10^{-3}$	$9.6 \cdot 10^{-4}$	$4.2 \cdot 10^{-3}$	$1.6 \cdot 10^{-3}$

Supplementary Table 7. System setup for the free energy of solvation calculations to validate the ligand models. Solvents are hexadecane (HD), octanol (OCO) and water (W). Note that we use hydrated OCO, i.e., we add a 0.35 mole fraction of W similar to experimental conditions.[2]

	T4 lysozyme	$A_{2A}R$	β2AR / FXR	*Vf*-ATA
ligands	benzene, phenol, indole, n-propylbenzene, thieno-pyridine, toluene, ethylbenzene	caffeine, ribose, adenine	adrenaline, propranolol / obeticholic acid	acetophenone
box size water (x×y×z) [nm^3]	5.5 × 5.5 × 5.5	5.5 × 5.5 × 5.5	4.3 × 4.3 × 4.3	7.0 × 7.0 × 7.0
number of CG water	1,356	1,356	700	2,703
box size HD (x×y×z) [nm^3]	5.3 × 5.3 × 5.3	5.3 × 5.3 × 5.3	4.3 × 4.3 × 4.3	—
number of CG HD	318	318	173	—
box size OCO (x×y×z) [nm^3]	5.3 × 5.3 × 5.3	5.3 × 5.3 × 5.3	4.3 × 4.3 × 4.3	7.0 × 7.0 × 7.0
number of CG OCO/W	664 / 58	664 / 58	311 / 28	1,332 / 116

Supplementary Table 8. Partitioning and SASA data for the ligands used in the present work as Martini 3 open-beta models. The free energy relative to the transfer of the solute molecule from solvent S1 to S2 ($\Delta G_{S1\rightarrow S2}$) obtained from experiments and computed at the CG level are shown. Solvents are hexadecane (HD), octanol (OCO) and water (W), which are described by a C1-C1-C1-C1, SC2-SC2-SP1, and W model, respectively. All the free energies are in kJ/mol. Statistical uncertainty for the computed ΔG is below 0.3 kJ mol^{-1} in all cases. Experimental data are from references [3,4].

		**$\Delta G_{OCO\rightarrow W}$ (kJ/mol)**			**$\Delta G_{HD\rightarrow W}$ (kJ/mol)**			**SASA (nm^2)**		
**molecule**	**CG model**	**Exp.**	**CG**	**Err.**	**Exp.**	**CG**	**Err.**	**AA**	**CG**	**Err. (%)**
benzene	TC4-TC4-TC4	12.1	14.2	-2.1	12.3	10.7	1.6	2.98	2.81	-5.7
phenol	TC4-TC4-SN3	8.3	4.3	4.0	-6.2	-5.2	-1.0	3.13	2.99	-4.6
n-propylbenzene	$(TC4)_3$-SC2	21.4	23.4	-2.0	22.1	22.7	-0.6	3.93	3.56	-9.4
acetophenone	$(TC4)_3$-SN2a	9.0	9.2	-0.2	—	—	—	3.69	3.42	-7.3
indole	$(TC4)_4$-T3Nd	12.2	11.0	1.2	—	1.9	—	3.50	3.43	-1.9
toluene	SC3-TC4-TC4	15.6	17.0	-1.4	15.3	16.4	-1.1	3.31	3.01	-8.8
ethylbenzene	$(TC4)_3$-TC3	18.0	19.9	-1.9	18.2	16.8	1.4	3.62	3.30	-8.7
thieno[2,3-c]pyridine	TC5-$(TC4)_3$-TN3a	9.9	9.7	0.2	—	-0.7	—	3.47	3.51	1.1

Supplementary Table 9. Partitioning and SASA data for the ligands used in the present work as models of a later development version of Martini 3. The free energy relative to the transfer of the solute molecule from solvent S1 to S2 ($\Delta G_{S1\rightarrow S2}$) obtained from experiments and computed at the CG level are shown. Solvents are hexadecane (HD), octanol (OCO) and water (W), which are described by a C1-C1-C1-C1, SC2-SC2-SP1, and W model, respectively. All the free energies are in kJ/mol. Statistical uncertainty for the computed ΔG is below 0.3 kJ mol^{-1} in all cases. Experimental data are from references [3,5,6].

		$\Delta G_{OCO\rightarrow W}$ (kJ/mol)			$\Delta G_{HD\rightarrow W}$ (kJ/mol)			SASA (nm^2)		
**molecule**	**CG model**	**Exp.**	**CG**	**Err.**	**Exp.**	**CG**	**Err.**	**AA**	**CG**	**Err. (%)**
caffeine	$(TN1)_3$-$(TP1a)_2$-TN5a-TC5	-0.4	1.4	-1.8	—	-19.3	—	4.41	4.26	-3.4
ribose	TP1-SP2-SP3	-13.3	-15.5	-2.2	—	-39.1	—	3.52	3.55	0.1
adenine	TN1-TN3a-TN5a-TP1d-TN3a-TC6	-0.5	-3.8	-3.3	—	-19.8	—	3.43	3.61	5.2
adrenaline	SQ4p-SP1-$(TC5)_2$-$(TP1)_2$	-7.8	-9.0	1.2	—	-32.7	—	4.64	4.66	0.4
propanolol	Q4p-SP1-TN2a-(TC5e)2-$(TC5)_3$	19.9	16.9	-3.0	—	3.9	—	6.18	6.33	2.4
chenodeoxy cholic acid	SP1-$(SC2)_2$-$(SC3)_2$-$(SC2)_2$-SQ5n	23.7	22.0	-1.7	—	14.4	—	7.72	7.38	-4.4
dasatinib	TP1-TN1-$(SC4)_2$-(TN1-SN4a-TN2d)-(TN6a-TC6-TC5)-SP2-(SX3-TC5-SC4)	—	—	—	—	—	—	9.00	8.76	-2.7
baricitinib	(TN5d-TC5-TC5)-$(TN6a)_2$-(TC5-TC5-TP2a)-(TC5-TN2a)-SN1-SP5a-TC3	—	—	—	—	—	—	6.66	6.52	-2.1

S3. SUPPLEMENTARY METHODS

Simulation settings. All simulations were performed with the program package GROMACS[7], version 2016.x or 2018.x. The coarse-grained (CG) force field Martini 3 (open-beta version or more recent development versions)[8] was employed for all simulations. Details about the generation of the protein models and the respective Martini version are given in the Methods Section of the manuscript. The ligand models are discussed in the Methods Section of the manuscript; their validation later in this Section.

The simulation parameters were chosen in accordance to reference [9]. The detailed simulation parameters for all CG simulations discussed in the present work are listed in Supplementary Table 4. Additional settings for the validation of the ligand models are given in Supplementary Table 5.

System setup. Supplementary Table 6 lists the setup of the different systems. In case of the A_{2A} receptor, one protein was initially placed in a lipid bilayer using the program *insane.py.*[10] The ligands were randomly placed in the water phase before solvating the system. The setup for the calculation of the free energy of solvation is given in Supplementary Table 7.

T4 lysozyme CG model. The CG model of the L99A single and the L99A/M102Q double mutant of T4 lysozyme was built using the open-beta version of the Martini 3 force field and the crystal structure with the pdb code 181L.[11] Although it contains the ligand benzene in its binding pocket, the structure is very similar to the one without any ligand (pdb code 4W51[12]) with an overall Cα RMSD of 0.2 Å. For the residues of the binding pocket, which were used for the alignment before the ligand RMSD calculation, the RMSD between both structures is with 0.2 Å very low as well.

The elastic network was set up using a force constant of 500 kJ/(mol·nm^2) and a distance cutoff of 0.9 nm.

A_{2A} receptor CG model. The CG model of adenosine A_{2A} receptor (A_{2A}R) was built using a later development version of the Martini 3 force field and the crystal structure with the pdb code 3RFM.[13] The mutations were reverted to the wild type and the missing loop, namely the extracellular loop 2 (ECL2), was modeled using the pdb structure 2YDO as reference.[14] The elastic network was set up using a force constant of 500 kJ/(mol·nm^2) and a distance cutoff of 0.8 nm. Because G-protein coupled receptors (GPCRs) are very flexible and can adapt to their environment and bound ligand,[15–19] a shorter distance cutoff for the elastic network was applied compared to the default value of 0.9 nm. The core and the binding pocket of A_{2A}R are considerably hydrated. To mimic the effect of the hydration, a tiny CG water bead (representing one to two water molecules) was placed inside the binding pocket. To represent these water molecules which are particularly important for ligand binding, regular CG water beads are too large. Thus, a tiny water bead was employed. It was kept in place via weak harmonic bonds to Asn181$^{5.42}$ and His250$^{6.52}$ using force constants of 500 kJ/(mol·nm^2) without excluding the non-bonded interactions. Additional weak harmonic bonds were added between the residues Thr88-Tpr246 and Asn181$^{5.42}$-His250$^{6.52}$ to include the effect of hydrogen bonds mediated by single water molecules in the binding pocket.[20] Again, force constants of 500 kJ/(mol·nm^2) were used without excluding the non-bonded interactions. Overall, these modifications stabilize the binding pocket. The protonation states of the residues were checked using the H++ server.[21] In accordance with the obtained predictions, Glu13 and His278 were considered charged. Asp52 was kept neutral as it is

quite buried in the protein core and experiments point to a Na^+ ion in close proximity in the case that the residue is charged which would compensate the negative charge.[20]

Adrenergic β2 receptor CG model. The CG model of adrenergic β_2 receptor (β2AR) was built using a later development version of the Martini 3 force field and the crystal structure with the pdb code 6MXT.[22] The mutations were reverted to the wild type, while the missing residues belonging to the intracellular loop 3 (ICL3) were modelled via MODELLER[23] using the pdb structure 3SN6[24] as reference. The secondary structure of this fragment of the protein was confirmed using prediction tools PSIpred[25] and Spider3.[26] The elastic network was set up using a force constant of 500 kJ/(mol·nm^2) and a distance cutoff of 0.8 nm. Due to the disordered nature of ICL3, no elastic network was added between the beads belonging to this part of the receptor. The intramolecular interactions of the binding pocket of β2AR were modelled based on ref. [27], as to stabilize the core of the molecule itself. Like for A_{2A}R, a tiny CG water bead was placed inside the core of the adrenergic receptor and kept in place via weak harmonic bonds to Asn293$^{6.55}$, Thr195^{ECL2} and His296$^{6.58}$ (superscripts refer to the Ballesteros-Weinstein numbering).[28] To mimic the internal hydrogen bond network described by Kobilka and co-workers,[27] additional weak bonds were added between residues Asp113$^{3.32}$-Tyr316$^{7.43}$, Thr118$^{3.37}$-Ser207$^{5.46}$, Ser204$^{5.43}$-Asn293$^{6.55}$ and Asn293$^{6.55}$-Tyr308$^{7.35}$. Again, force constants of 500 kJ/(mol·nm^2) were used without excluding the non-bonded interactions. The protonation states of the residues at pH 7.0 were checked using the H++ server.[21] In accordance with the obtained predictions, His296$^{6.58}$ was considered charged. In addition, Glu122$^{3.41}$ and Asp79$^{2.50}$ were kept neutral, following Dror et al..[29]

Farnesoid X receptor CG model. Generation of the CG model of farnesoid X receptor (FXR) was performed using a later development version of the Martini 3 force field and the crystal structure with pdb code 1OSV.[30] This protein structure belongs to Rattus Norvegicus, but shares a very high identity with the human FXR, as high as 95%. For these reasons, it has been employed in several pharmacological investigations.[31–33] At variance with the other receptors, in this case the elastic network was assembled using a force constant of 1000 kJ/(mol·nm^2) and a distance cutoff of 0.75 nm. The slightly shorter cutoff and doubled force constant were needed to prevent the beads from becoming too "sticky" and in turn the binding pocket from collapsing.[34] To properly take into account for the relatively high flexibility of some regions of FXR, such as helices 11 and 12, or the loop connecting helices 5 and 6, several rubber bands were removed to make the model less rigid. The flexibility of the CG model was compared against the data obtained from a long all-atom simulation (AA)[32] performed using the Amber99SBildn force field.[35] Four additional bonds with a force constant of 1000 kJ/(mol·nm^2) were added between residues Ala288-Met325, Ala288-Ser329, Val292-Ala321 and Val292-Met325 to reproduce the conformational freedom of the binding pocket observed in the AA simulation. The protonation state of the residues was assigned accordingly to the most predominant form at pH 7.0, as computed by the H++ server.[21] Consequently, His426 was considered charged. His444 was considered charged too, as to replicate the cation-π interaction formed with Trp466.[31–33]

c-Src kinase CG model. The CG model of the kinase domain was built using a later development version of the Martini force field and the crystal structure with the pdb code 1Y57 as reference (residues 290–533).[36] The crystal structure contains the c-Src complex in active conformation bound to a des-methyl analog of the drug imatinib. Protonation of His, Lys, Glu, Arg, and Asp

residues close to the binding site was defined in accordance to predicted pKa values.[37] An elastic network was employed to keep the secondary and tertiary structure of the protein with a force constant of 700 kJ/(mol·nm^2) and a distance cutoff of 0.9 nm. Comparison of the RMSD of the backbone beads in CG simulations and the RMSD of Cα from atomistic reference simulations (Amber ff14SB force field)[38] shows a good agreement.

***Vibrio fluvialis* aminotransferase CG model.** The CG model of *Vf*-ATA was built using the open-beta version of the Martini 3 force field and the crystal structure with the pdb code 4E3Q as reference.[39] The crystal structure contains the cofactor pyridoxamine phosphate (PMP) but no substrate is present in the binding pocket. An elastic network was employed to keep the secondary and tertiary structure of the protein with a force constant of 500 kJ/(mol·nm^2) and a distance cutoff of 0.9 nm. No distance constraints were employed to maintain the quaternary structure of the homodimer in the CG simulations. Comparison of the pairwise distances between the Cα atoms of the two monomers with distances from atomistic reference simulations shows a good agreement. One cofactor PMP was added to each of the two binding pockets and bound to the protein by harmonic bonds.

AAK1 CG model. The CG model of the AP2-associated protein kinase 1 (AAK1) was built using a later development version of the Martini 3 force field and the crystal structure with the pdb code 5L4Q.[40] The elastic network was set up using a force constant of 500 kJ/(mol·nm^2) and a distance cutoff of 1.0 nm. The protonation states of the residues were chosen in accordance with the predictions from the H++ server.[21] Due to the lack of a crystal structure reference of baricitinib bound to AAK1, we used the structure of the homolog BMP-2-inducible kinase bound to

baricitinib (pdb code: 4W9X[41]) to model the binding mode of baricitinib. Overall, the BMP-2-inducible kinase has 72% of identity with AAK1; the catalytic site is fully conserved (100% identity), which makes it a good reference for the baricitinb binding mode to AAK1. Baricitinib binding pose and the missing residues in AAK1 pdb structure were modelled by sequence and structural alignment with BMP-2-inducible kinase.[42]

Ligand CG models. Benzene, toluene, and phenol are described by three T-bead models, TC4-TC4-TC4 in the case of benzene, while toluene and phenol by a TC4-TC4-SC3 and TC4-TC4-SN3 models, respectively. In the latter two cases, the S-bead used accounts for the extra non-hydrogen atom. Ethylbenzene, n-propylbenzene, and acetophenone are all described by four-bead models containing the benzene moiety and a substituent: this leads to TC4-TC4-TC4-TC3, TC4-TC4-TC4-SC2, and TC4-TC4-TC4-SN2a, were the TC3, SC2, and SN2a beads represent the ethyl, n-propyl, and ethanoyl groups in the three molecules, respectively. Lastly, indole and thieno[2,3-c]pyridine are described by five-bead models $(TC4)_4$-TN3d and TC5-$(TC4)_3$-TN3a, respectively. A representation of the models on top of the underlying atomistic structures is shown in Supplementary Fig. 1. Comparison between CG and reference partitioning, molecular volume, and mass density data are shown in Supplementary Tables 8 and 9. They show an overall good agreement between CG and reference properties.

The indole model uses a virtual site to improve the numerical stability of the model.[43] In particular, the model exploits the “hinge” construction – inspired by the one used in the latest cholesterol model of Melo and co-workers[44] – for the four external beads while the central bead is described as a virtual site, *i.e.*, a particle whose position is completely defined by its constructing particles (in this case, the other four beads). Such a model not only improves the numerical stability of the

model but also its performance due to the use of fewer linear constraints. The caffeine model uses the same "hinge" construction; four beads are used to build the hinge while the remaining three beads are described with virtual sites.

The bonded parameters of benzene, toluene, ethyl-benzene, n-propyl-benzene, phenol, indole, thieno[2,3-c]pyridine, caffeine, dasatinib, and baricitinib were obtained using as reference atomistic trajectories obtained using the OPLS-AA force field, with parameters obtained via the LigParGen server.[45–47] In some cases (toluene, ethyl-benzene, n-propyl-benzene, and thieno[2,3-c]pyridine), the final bonded parameters of the CG models were slightly modified to better match the atomistic solvent accessible surface area values. To obtain the bonded parameters of acetophenone, distributions of distances, angles and dihedrals from an atomistic 100 ns simulation of acetophenone in water using the AMBER03 force-field were used as target.

The CG structure of 6-ethyl-chenodeoxycholic acid (6-ECDCA, obeticholic acid) was developed based on the cholesterol model of Melo and co-workers,[44] due to their high similarity. The mass, type, and position of the beads were adjusted as to take into account the different conformations of the molecules, the extra hydroxyl group, the ethyl group in position 6α, the shorter alkyl chain and the carboxyl group situated in position 5β.

To the best of our knowledge, no experimental data is available regarding the partitioning free energy of obeticholic acid. Since validation of the model would have been difficult without this information, we turned our attention to chenodeoxycholic acid (CDCA). Its structure is extremely similar to the one of obeticholic acid, the only difference being the lack of the ethyl group on the β ring. Therefore, we assembled a CG model of CDCA by removing the SC3 bead representing the ethyl group of obeticholic acid to compare its partitioning free energy with the available experimental data, which served as cross-validation of the obeticholic acid model.

The propanolol model uses the "hinge" construction for representation of the napthalene ring, which is built using three TC5 beads, one being described as virtual site, and two TC5e beads, which were introduced in the re-parametrized Martini force field to properly represent the electron density of such aromatic rings. The distributions of distances, angles and dihedrals for 6-ECDCA, CDCA, adrenaline and propranolol were derived from atomistic 100ns-long simulations of each molecule solvated in water. The AA models of the ligands were developed using the GAFF2 force field.[48]

Ligand model validation. Thermodynamic integration (see Supplementary Table 5 for detailed settings) was used to compute solvation free energies ($\Delta G_{\text{Ø}\rightarrow S}$) in different solvents, using the Multistate Bennett Acceptance Ratio (MBAR) to get the final results and associated errors.[49] The free energy associated with transferring a solute from a solvent S1 to a solvent S2 ($\Delta G_{S1\rightarrow S2}$) was computed as the difference $\Delta G_{S1\rightarrow \text{Ø}} - \Delta G_{S2\rightarrow \text{Ø}}$. Results are shown in Tables S5 and S6. Solvent accessible surface area (SASA) values were computed with the GROMACS [7] tool *gmx sasa* using the following command line:

gmx sasa -s molecule.pdb -o sasa.xvg -probe 0.185 -ndots 4800

A *molecule.gro* file can also be used instead of the *.pdb* file. The size of the probe corresponds to the radius of a tiny (T) bead. The probe size impacts the absolute SASA values, but not their relative difference. So, for comparing SASA values, the same probe size has to be used. The flag *-ndots* specifies the accuracy of the calculation: 4800 (or higher) was found to be necessary for accurate SASA computations.

RMSD and ligand densities of T4 lysozyme. To calculate the RMSD between the simulated binding poses and the crystal structure shown in Fig. 1A of the manuscript, the binding pocket was aligned to the CG crystal structure. We used the following residues for the alignment: Ile78 (SC1); Leu84 (SC1); Val87 (SC1); Tyr88 (BB); Ala99 (BB); Met102 (SC1); Val111 (SC1); Phe153 (SC1); Leu118 (SC1). The specific CG beads are given in parenthesis. Finally, before the RMSD calculation, the CG ligand in the crystal structure, which was obtained by transforming the bound atomistic ligand to its CG resolution, was minimized for one step to account for slight changes in the bonded parameters. Note that in case of ligands with high symmetry like benzene, all possible orientations have to be taken into account. The lowest RMSD of all possible orientations is the correct value because all other values are too high due to flipping or rotation of the structure which does not change the chemical structure of the binding pose.

To generate the ligand densities around L99A T4 lysozyme shown in Fig. 1 of the manuscript, first, the ligand was placed at the minimum distance to the protein in every snapshot with respect to the periodic boundary conditions. Then the protein was positioned in the box center and its backbone was aligned with the crystal structure. The ligand density was obtained by computing the occupancy of the ligand in the three-dimensional space using the Volmap plugin of VMD.[50] The grid points had a distance of 0.2 nm. All ligand beads were taken into account in their actual size: The radius of small beads is 0.23 nm; the one of tiny beads 0.19 nm. Similarly, the ligand densities depicted in Supplementary Fig. 2 were calculated.

RMSD of A_{2A} receptor. To calculate the RMSD of the ligand binding, we used the ligand and its contact beads in the crystallographic structure as reference. We considered all the residues whose distance from the ligand was less than 7 Å as contact beads. In the case of adenosine, the backbone

beads (BB) of the following residues were used to align the protein before the RMSD calculation: Ala59, Ala63, Ile66, Val84, Leu85, Thr88, Phe168, Glu169, Val172, Met174, Met177, Asn181, Trp246, Leu249, His250, Asn253, Met270, Ile274, Ser277, and His278. In the case of caffeine, the backbone beads (BB) of Tyr9, Ala63, Ile66, Ser67, Val84, Leu85, Thr88, Phe168, Glu169, Met177, Leu249, His250, Asn253, Met270, Ile274, Ser277, and His278 were aligned. Finally, before the RMSD calculation, the CG ligand in the crystal structure, which was obtained by transforming the bound atomistic ligand to its CG resolution, was minimized for one step to account for slight changes in the bonded parameters.

RMSD of adrenergic β2 receptor. The RMSD of the ligand binding was computed using as reference the crystallographic pose (pdb code 4LDO[27] for adrenaline and 6PS5[51] for propanolol). Ligand and contact beads, i.e. all the beads located at less than 7 Å of distance from the ligand, were employed in RMSD calculations. The residues used for aligning the adrenaline-β2AR complex before computing the RMSD are the following: Asp113$^{3.32}$, Val114$^{3.33}$, Val117$^{3.36}$, Thr118$^{3.37}$, Phe193^{ECL2}, Tyr199$^{5.38}$, Ser203$^{5.42}$, Ser204$^{5.43}$, Ser207$^{5.46}$, Trp286$^{6.48}$, Phe289$^{6.51}$, Phe290$^{6.52}$, Asn293$^{6.55}$, Asn312$^{7.39}$, Tyr316$^{7.43}$. Only the backbone beads (BB) were employed. In the case of propanolol, the same beads were used for alignment, plus the BB bead of Ala200$^{5.39}$. Finally, the CG ligand in the crystal structure, obtained by mapping the atomistic molecule to its CG model, was minimized for one step to account for slight changes in the bonded parameters before the RMSD calculation.

Binding simulations for β2AR. A second batch of MD calculations was performed to simulate the desolvation of the β2AR binding pocket. From the *pre-binding simulations* all the poses having

a RMSD lower than 5 Å were extracted and clustered via the *gmx cluster* tool. The centroids of the first 12 clusters, corresponding to ~ 85% of the observed binding poses, were used as starting point for additional simulations. Each structure was replicated thrice and simulated for 100 ns, for a grand total of 3.6 µs. Desolvation of the binding pocket was obtained by steering the water beads inside the binding site within 4 Å of Ser204$^{5.43}$, Trp286$^{6.48}$, Phe289$^{6.51}$, Asn293$^{6.55}$, Asn312$^{7.39}$, Tyr316$^{7.43}$ and Asp113$^{3.32}$, Val114$^{3.33}$, Val117$^{3.36}$, Thr118$^{3.37}$, Phe193^{ECL2}, Tyr199$^{5.38}$ using the *plumed* plugin.[52] To avoid ligand departure from the extracellular vestibule prior the desolvation of the binding site, its movement was kept within a distance of 9 Å computed between its center of mass and the backbone bead of Asn293$^{6.55}$.

RMSD of farnesoid X receptor. The RMSD of the ligand binding was calculated with the same protocol employed for A$_{2A}$R and β2AR complexes, using the crystal with pdb code 1OSV as reference. The BB beads of the following residues were used for alignment of the complex before computing the RMSD: Met262, Leu284, Thr285, Met287, Ala288, His291, Met325, Arg328, Ser329, Ile359, Met362, His444.

RMSD and binding rate of c-Src kinase. For the calculation of the ligand RMSD, the crystal structure of an c-Src from chicken containing dasatinib (pdb code 3G5D[53]) was aligned to the structure of the human c-Src (pdb code 1Y57) (MUSTANG method,[54] RMSD of 0.61 Å over 226 aligned residues with 98.7% sequence identity). The resulting atomistic structure of dasatinib bound to the binding site of 1Y57 was converted to a CG structure and minimized for one step to account for slight changes in the bonded parameters. The protein backbone was used for alignment of the CG trajectory (for Fig. 5B the alignment was done using the backbone atoms of residues

273 274 281 293 295 323 336 338 339 340 341 344 345 393 403 404 composing the active site). The CG structure of the ligand and the contact protein beads were then compared to the CG model of the substrate bound to the active site to obtain their RMSD.

The binding rate of dasatinib was estimated following the procedure of Shan et al.:[55]

$$k_{on} = \frac{\frac{number\ of\ binding\ events}{simulation\ time}}{[concentration]} \quad (1)$$

RMSD and stereoselectivity of *Vibrio fluvialis* aminotransferase. For the calculation of the ligand RMSD, the QM/MM optimized structure of the cofactor-bound substrate was aligned with the crystal structure to serve as a reference. The heavy atoms of the cofactor were used for the alignment. To the best of our knowledge, no crystal structure with acetophenone present in the binding pocket is currently available. The resulting atomistic structure of acetophenone was converted to a CG structure as described in the Methods Section of the manuscript and minimized for one step to account for slight changes in the bonded parameters. The protein backbone was used for the alignment of the CG trajectory. The CG structures of the ligand and the contact protein beads were then compared to the CG model of the substrate bound in the active site to obtain their RMSD.

The active site of *Vf*-ATA has a large and a small binding pocket which is important to achieve the high stereoselectivity during the reaction. To estimate in how many binding events the large substituent of acetophenone (phenyl ring) is correctly positioned in the large binding pocket and how often it is positioned in the small binding pocket, the density of the CG bead representing the *p*-C atom with respect to the carbonyl group was calculated. The ratio of the *p*-C atom density in the large pocket versus the small one is 3:1. Thus, the ligand is positioned correctly in 75% of the binding events. However, because the stereoselective reaction step occurs not directly after the

substrate binding to *Vf*-ATA,[1] a reorientation of the substrate could in principle still occur during the next reaction steps to achieve the high stereoselectivity.

RMSD of AAK1. To calculate the RMSD of the ligand binding, we used the ligand and its contact beads in the crystallographic structure as reference. We considered all the residues whose distance from the ligand was less than 5 Å as contact beads: Leu52 (SC1), Ala53 (BB), Val60 (SC1), Ala72 (BB), Val104 (SC1), Met126 (BB and SC1), Asp127 (BB), Phe128 (BB, SC1, and SC2), Cys129 (BB and SC1), Glu180 (BB), Asn181 (BB and SC1), Leu183 (SC1), Cys193 (SC1), and Asp194 (SC1). Before the RMSD calculation, the beads of the pocket were aligned. In addition, the CG ligand in the crystal structure, which was obtained by transforming the bound atomistic ligand to its CG resolution, was minimized for one step to account for slight changes in the bonded parameters. Ligand densities were computed using the same procedure described for T4 lysozyme.

S4. SUPPELEMENTARY DISCUSSION

Ligand densities around T4 lysozyme. Supplementary Fig. 2 shows the densities of the seven ligands phenol, thieno-pyridine, benzene, indole, toluene, ethylbenzene, and n-propylbenzene around the L99A single and L99A/M102Q double mutants of T4 lysozyme, respectively. If the density is for the double mutant, the ligand name is followed by the second mutation M102Q.

RMSD of the T4 lysozyme ligands. Supplementary Fig. 3 shows the RMSD distributions for the binding simulations of the four ligands benzene, phenol, indole, and n-propylbenzene to T4 lysozyme. While the distributions for benzene and phenol exhibit a reasonable Gaussian shape, the ones of indole and n-propylbenzene show two sub-populations. The corresponding fitting

parameters of the RMSD distributions using two Gaussian functions (blue line) are given in Supplementary Table 4. The green line depicts the fit using a single Gaussian function; the fitting parameters are given in Table 1 of the manuscript.

Details of the $A_{2A}R$ binding. The RMSD was computed using the ligand and its contact beads in the crystallographic structure as reference. We considered as contact beads all the residues whose distance from the ligand was less than 7 Å. The RMSD histogram has a peak at 3.1 Å, with an average of 3.3 Å and a standard deviation of 0.5 Å, whereas the best binding pose has an RMSD of 2.2 Å. Compared to the crystal,[14] in this pose adenosine is shifted toward transmembrane helix 7 (TM7), with a slight tilt of 14°. All the interactions observed in the crystallographic structure are here preserved: the purine ring is directly facing the side chains of residues Leu249$^{6.51}$, Ser277$^{7.42}$ and His278$^{7.43}$ (superscript refers to Ballesteros-Weinstein numbering[28]), which lie at 4 Å from the ligand. The orientation of the side-chains of Ser277$^{7.42}$ and His278$^{7.43}$ is such that they can establish hydrogen bond interactions with adenosine, as also reported in the crystallographic structure. Other important contact residues that are located within 5 Å from adenosine are Met270$^{7.35}$, Ile274$^{7.39}$, Asn253$^{6.55}$, Phe168^{ECL2}, Val84$^{3.32}$, Ala63$^{2.61}$, and Thr88$^{3.36}$. At higher values of distance, between 6 and 8 Å, we can find His250$^{6.52}$, Ile66$^{2.64}$, Trp246$^{6.48}$, Asn181$^{5.42}$, and Met177$^{5.38}$, the furthest ones being known for forming a network of hydrogen bonds with adenosine via water molecules. Overall, the best binding mode closely resembles the crystallographic one, especially in terms of interactions with key residues of the $A_{2A}R$ binding site.[14] This result is extremely positive, all the more so since only 15 binding events were observed, proving the robustness of the force field even with extremely complex systems.

More lenient was the case of the $A_{2A}R$ – caffeine system. Here, we observed 142 binding events, which led to a well sampled binding mode. Fig. 3B (bottom) of the manuscript shows the distribution of the RMSD of all binding poses, with an average of 3.4±0.5 Å and a peak at 3.3 Å. The best binding mode has a RMSD of 1.9 Å and is remarkably similar to the crystallographic one, being slightly shifted by 1.4 Å towards TM3 and tilted by 4°.[13] Caffeine is directly facing TM2 and TM3, just beneath ECL1, being only 4 Å apart from Phe168^{ECL2} and His278$^{7.43}$. Leu249$^{6.51}$ and Asn253$^{6.55}$ lie at 5 Å, whereas Ser277$^{7.42}$ and Ile274$^{7.39}$ are situated at 6 Å of distance from the ligand. His278$^{7.43}$ is oriented toward the bead representing one of the two carboxylic groups of the ligand in a competent position to establish a hydrogen bond interaction as reported in the crystallographic structure.[13] Lastly, Val84$^{3.32}$, Met177$^{5.38}$, Asn253$^{6.55}$, Thr88$^{3.36}$, Trp246$^{6.48}$, and Met270$^{7.35}$ are all situated nearby the ligand able to form Van der Waals contacts. Even in this case, we find a good agreement with the crystal, notwithstanding the coarse-grained nature of the models and of the intermolecular interactions treatment.

We further analyzed our simulations to provide structural insight into the ligand binding mechanism. We remark once more that our simulations start with the ligand in the bulk solvent where no contact with the protein is present. In 104 out of 157 binding events observed for both adenosine and caffeine, the ligand firstly interacts with either ECL2 or ECL3 before going through the passage made by TM3, TM6 and TM7 and reaching the binding pocket (Fig. 3C and D of the manuscript). The initial stages of binding are marked by the interaction of adenosine with residues Lys150^{ECL2}, Lys153 ^{ECL2}, Ser156 ^{ECL2} and Gln157 ^{ECL2} (11 binding events out of 15), which are oriented toward the interior of the gorge (Fig. 3C, black solid line). In the remaining cases, the ligand instead firstly binds ECL3 by interacting with His264^{ECL3}, then points towards the entry of the binding pocket (Fig. 3C, red solid line). In this phase, the ligand-protein interaction is

strengthened by the formation of new contacts between adenosine and residues Tyr9$^{1.33}$, Ser67$^{2.65}$, Leu167^{ECL2} and Glu169^{ECL2}. Finally, the ligand reaches the upper portion of the binding pocket by interacting with Ile66$^{2.64}$, Phe168^{ECL2} and Asn253$^{6.55}$, thus entering the binding site. In contrast to adenosine, caffeine interacts with ECL3 and TM6 (Fig. 3D, green solid line, 57 binding events out of 142) or TM1 and TM7 in the initial binding stage, entering into the cleft formed by the latter two helices (Fig. 3D, black solid line, 51 binding events out of 142). The most recurrent interactions observed in this phase involve Tyr9$^{1.33}$, Thr256$^{6.58}$, Phe257$^{6.59}$, His264^{ECL3}, Leu267$^{7.32}$ and Met270$^{7.35}$. In other cases (32 binding events out of 142), the ligand binds A$_{2A}$R through ECL2, directly interacting with Leu167^{ECL2} and Glu169^{ECL2} (Fig. 3D, red solid line). It is worth mentioning that from the very beginning, caffeine shows a tendency to interact with residues close to the entry of the binding pocket. Like for adenosine, entry inside the binding pocket is forerun by the establishment of contacts with residues Ser67$^{2.65}$, Phe168^{ECL2}, Met174$^{5.35}$ and Asn253$^{6.55}$.

Details of the β2AR binding. Adrenergic β$_2$ receptors (β2ARs) are rhodopsin-like receptors and, similarly to A$_{2A}$R, have been deeply investigated in the last decades due to their role in regulating smooth muscle relaxation and heart rate.[56,57] Together with A$_{2A}$R, β2AR represents a prime model for studying the activation mechanism of GPRCs, thanks to the plethora of crystallographic structures available in the inactive, intermediate and active conformations.[56] In the present work, we simulated the binding to β2AR of one natural agonist, adrenaline, and one inverse agonist, propanolol.

Similarly to A$_{2A}$R, β2AR is endowed with a narrow binding pocket. At variance with the adenosine receptor, ligand binding to β2AR has been reported to occur through a 2-step mechanism; first the ligand approaches the extracellular vestibule under the extracellular loop 2

(ECL2) and then reaches the final binding site passing through two gating residues: Phe193^{ECL2} and Tyr308$^{7.35}$ (superscripts refer to the Ballesteros-Weinstein numbering).[28,29] This passage requires the desolvation of both the binding site and the ligand, as shown in a computational study.[29] As such, β2AR represents a more challenging case than $A_{2A}R$ in which we probe the capability of the Martini 3 force field to reproduce the crystallographic binding poses of adrenaline and propanolol.

The two systems (β2AR – adrenaline and β2AR – propranolol) were simulated for an overall time of 240 µs each one (12 simulations with a length of 20 µs each, hereinafter referred as *pre-binding simulations*). The simulation box contains a β2AR protein embedded in a POPC bilayer and 10 ligands, either adrenaline or propranolol, randomly placed in the unbound state. After the end of the calculations, all the poses having an RMSD lower than 5 Å were extracted and clustered. The centroids of the first 12 clusters, representing more than 85% of the observed binding poses, were further simulated for 300 ns during which the binding site was desolvated (hereinafter referred as *binding simulations*) (see Supplementary Methods for details). The binding poses were compared with crystal structures 4LDO[27] for adrenaline and 6PS5[51] for propanolol.

Supplementary Fig. 5B shows the RMSD distribution of all the observed binding poses of adrenaline. The red bars refer to the prebinding simulations, whereas the black ones are related to the binding simulations. The best binding pose has a RMSD of 1.0 Å and it is shown in orange in Supplementary Fig. 5D. Such pose is almost identical to the crystallographic one, with adrenaline slightly rotated of 14° towards helix 3. The tail of adrenaline assumes the same orientation of the crystallographic one.

The RMSD plot of the ligand relative to the X-ray pose in the *prebinding simulations* is reported in Supplementary Fig. 5B (red bar) and clearly shows the presence of the vestibular extracellular

binding site. In fact, in these simulations the majority of the bound conformations belong to a family at 4.4 Å of RMSD in which adrenaline has penetrated inside the extracellular vestibule previously identified by Dror et al.[29] Only after the desolvation of the binding site in the *binding simulations*, adrenaline is able to leave the extracellular vestibule and enter the binding pocket, passing through the gate formed by Phe193^{ECL2} and Tyr308$^{7.35}$. In these simulations (black bars in Supplementary Fig. 5B) the two most populated binding conformation families at 2.5 Å and 1.6 Å of RMSD show adrenaline inside the binding pocket with a binding mode very similar to the crystallographic one.

A similar behavior is observed in the case of propanolol (Supplementary Fig. 5C). The *pre-binding simulations* show an RMSD peak at 4.3 Å where the ligand is bound to the extracellular vestibular binding site. Here, the presence of several water beads (between 2 and 4, corresponding to 8 and 16 atomistic water molecules) inside the binding site impedes the ligand to reach the final binding mode. As seen for adrenaline, even in this case in the *binding simulations* the ligand deepens in the receptor reaching the final binding mode only when the water molecules leave the binding pocket. In these simulations, we find a first peak at 3.4 Å and two smallest peaks at 2.3 Å and 1.5 Å. The best bound pose (Supplementary Fig. 5E, in orange) has a RMSD value of 0.9 Å, being almost identical to the one observed in the x-ray structure with a tilt of 31° towards helix 4 and a slightly different orientation of the tail.

It is interesting to note that the binding mechanism is different between adrenaline and propranolol. In the case of adrenaline, in all the observed 40 binding events the ligand enters the extracellular vestibule from the water phase by interacting with ECL2 (Supplementary Fig. 5D, black and red solid line). In 18 events, the ligand also interacts with ECL3 and moves on the surface of the extracellular vestibule, before stopping at the gate (Supplementary Fig. 5D, red solid

line). On the other hand, in the majority of the propanolol binding events (5/7), the ligand approaches β2AR from the lipid bilayer (Supplementary Fig. 5E, black and red solid line) and then reaches the gate by passing through the cleft formed by the N-terminal ends of helix 1 and helix 7 (Supplementary Fig. 5E, black solid line, 4/7 events) or passing over ECL1 (Supplementary Fig. 5E, red solid line, 1/7 events). In the two remaining binding events, propranolol follows a pathway similar to the ones observed for adrenaline, reaching the extracellular vestibule from the water phase by interacting with ECL2 (Supplementary Fig. 5E, green solid line). The different behavior of the ligands in approaching the receptor might depend on the different polarity of the ligands as also observed in the case of adenosine and caffeine in $A_{2A}R$.

Details of the FXR binding. Due to minor conformational changes of the binding site of FXR when passing from the apo (unbound) to the holo form (ligand bound) (see Fig. 3E in the manuscript), the solvent accessible surface area (SASA) of the binding pocket (excluding the ligand in the calculation) increases from the apo to the holo form (Supplementary Fig. 6, blue solid line).

Details of the S-selective aminotransferase binding. As an additional showcase for ligand binding to an enzyme, we show the S-selective aminotransferase (ATA) of *Vibrio fluvialis* which stereoselectively catalyzes the transfer of an amino group from a donor to an acceptor ketone.[1] Due to the high stereoselectivity of the reaction, ATAs are of particular interest for the synthesis of pharmaceutical compounds. This second example is more challenging, because the active site has a small and a large pocket in which the substrate needs to be properly positioned to achieve the high stereoselectivity.[58] We use acetophenone as substrate which is commonly used to study

ω-ATAs. The phenyl and the methyl group of acetophenone bind to the large and the small binding pocket, respectively, which is important for the stereoselectivity. Because *Vf*-ATA is a homodimer with two active sites, we include ten substrate molecules in a cubic simulation box with 16 nm edge length (Supplementary Fig. 8A). The protein is solvated in ~33,000 CG water beads, resulting in a substrate concentration of 4.2 mM which is comparable to the concentration used in experimental studies.[59,60] Supplementary Fig. 8C depicts the binding path of the center of mass of acetophenone during one of the observed binding events that took place within 6 ns. The binding path shows that the substrate is capable of entering the 15 Å deep tunnel to the active site during unbiased Martini simulations. Supplementary Fig. 8B (left) shows the acetophenone density in the *Vf*-ATA binding pocket (transparent red isosurface). In addition, several CG snapshots of acetophenone (red) taken from our binding simulations and the acetophenone binding pose in a DFT optimized binding site (green)[1] are depicted. They show a good agreement with an average RMSD of < 4 Å (Supplementary Fig. 8B, right). A closer look at the binding path reveals that acetophenone is in contact with the residues Arg415 and Phe86 while exploring the tunnel towards the active site. Both amino acids are routinely mutated to tailor the enzyme activity of *Vf*-ATA.[58,59,61] An estimate, based on the density of the CG bead representing the *p*-C atom with respect to the carbonyl group, yields a correct positioning of the phenyl substituent in the large binding pocket in approximately 75% of the bound structures. Compared to T4 lysozyme, binding and unbinding events happen more often in *Vf*-ATA with about three bindings per μs and active site. This higher frequency of binding and unbinding is expected for an enzyme in order to have a minimum of lag time between two catalytic turnovers. Typical association rate constants for enzymes are in the order of 10^6-10^9 $M^{-1}s^{-1}$,[62] and a few known values for the formation of the enzyme-substrate Michaelis complex in ATAs lie in the range of 10^5-10^8 $M^{-1}s^{-1}$.[63–65]

Computational performance. The computational performance in relation to all-atom models was evaluated considering two aspects: (i) performance of the MD code; (ii) speed up due to smoother potential landscape of CG resolution. Benchmark performance tests were performed with the program package GROMACS 2018,[7] using a HP Workstation Z4, with Intel® Xeon® W-2135 Processor and GP102 GeForce GTX 1080 Ti. Short 5 ns MD simulations were performed using the default domain decomposition options from GROMACS, using the GPU and a specific number of CPUs, depending of the size of the system (see Supplementary Table 2). Built with CHARMM-GUI,[66] the smallest system was $7.6 \times 7.6 \times 7.6$ nm^3 box of T4 lysozyme in 0.15 M NaCl water solution. This box was replicated 8 and 16 times, building bigger systems with the same protein and salt concentration. Equivalent coarse-grained systems were built with Martini 3. The CHARMM all-atom force-field[67] was used for comparison with Martini 3. The results are displayed in Supplementary Table 2. Overall, these benchmarks showed that Martini can be 110–350 times faster than all-atom simulations, with the performance gain increasing with increasing system size.

Calculations of the diffusion coefficient in water (D_w) at 298 K were performed for 5 small-molecules: ethylbenzene, cyclohexane, toluene, phenol and benzene. One solute molecule was placed in a water cubic box of $6.8 \times 6.8 \times 6.8$ nm^3. MD simulations were performed for 4.8 μs, with D_w computed from linear regression of the mean-squared displacement, fitted between times 0 to 100 ns. The idea was to obtain an estimate of the speed-up due to the smoother potential landscape of the Martini 3 CG models. Considering that atomistic models can reproduce reasonably well diffusion coefficients, the results of Martini MD simulations were compared with experimental data,[68] as shown in Supplementary Table 3. Martini 3 presented diffusion coefficients

were 2.9 ± 0.2 times higher than the experimental data. Trends in D_w seem to be reasonably good (R^2=0.82).

S5. REFERENCES

1. Cassimjee, K. E., Manta, B. & Himo, F. A quantum chemical study of the ω-transaminase reaction mechanism. *Org. Biomol. Chem.* **13**, 8453–8464 (2015).

2. Bernazzani, L., Cabani, S., Conti, G. & Mollica, V. Thermodynamic study of the partitioning of organic compounds between water and octan-1-ol. Effects of water as cosolvent in the organic phase. *J. Chem. Soc. Faraday Trans.* **91**, 649 (1995).

3. Abraham, M. H., Chadha, H. S., Whiting, G. S. & Mitchell, R. C. Hydrogen Bonding. 32. An Analysis of Water-Octanol and Water-Alkane Partitioning and the Δlog P Parameter of Seiler. *J. Pharm. Sci.* **83**, 1085–1100 (1994).

4. Natesan, S. *et al.* Structural Determinants of Drug Partitioning in *n* -Hexadecane/Water System. *J. Chem. Inf. Model.* **53**, 1424–1435 (2013).

5. Hansch, C., Leo, A. & Hoekman, D. *Exploring QSAR, Vol. 1: Fundamentals and applications in chemistry and biology*. (American Chemical Society, 1995).

6. Hansch, C., Leo, A. & Hoekman, D. *Exploring QSAR, Vol. 2: Hydrophobic, electronic, and steric constants*. (American Chemical Society, 1995).

7. Abraham, M. J. *et al.* GROMACS: High performance molecular simulations through multi-level parallelism from laptops to supercomputers. *SoftwareX* **1–2**, 19–25 (2015).

8. Souza, Paulo C.T., Marrink, S. J. Martini 3 - Open beta-release. Available at: http://cgmartini.nl/index.php?start=12. (Accessed: 3rd September 2019)

9. de Jong, D. H., Baoukina, S., Ingólfsson, H. I. & Marrink, S. J. Martini straight: Boosting performance using a shorter cutoff and GPUs. *Comput. Phys. Commun.* **199**, 1–7 (2016).

10. Wassenaar, T. A., Ingólfsson, H. I., Böckmann, R. A., Tieleman, D. P. & Marrink, S. J. Computational Lipidomics with *insane* : A Versatile Tool for Generating Custom

Membranes for Molecular Simulations. *J. Chem. Theory Comput.* **11**, 2144–2155 (2015).

11. Morton, A. & Matthews, B. W. Specificity of ligand binding in a buried nonpolar cavity of T4 lysozyme: Linkage of dynamics and structural plasticity. *Biochemistry* **34**, 8576–8588 (1995).

12. Merski, M., Fischer, M., Balius, T. E., Eidam, O. & Shoichet, B. K. Homologous ligands accommodated by discrete conformations of a buried cavity. *Proc. Natl. Acad. Sci. U. S. A.* **112**, 5039–44 (2015).

13. Doré, A. S. *et al.* Structure of the adenosine A(2A) receptor in complex with ZM241385 and the xanthines XAC and caffeine. *Structure* **19**, 1283–93 (2011).

14. Lebon, G. *et al.* Agonist-bound adenosine A2A receptor structures reveal common features of GPCR activation. *Nature* **474**, 521–525 (2011).

15. Escribá, P. V., Wedegaertner, P. B., Goñi, F. M. & Vögler, O. Lipid–protein interactions in GPCR-associated signaling. *Biochim. Biophys. Acta - Biomembr.* **1768**, 836–852 (2007).

16. Latorraca, N. R., Venkatakrishnan, A. J. & Dror, R. O. GPCR Dynamics: Structures in Motion. *Chem. Rev.* **117**, 139–155 (2017).

17. Sengupta, D., Prasanna, X., Mohole, M. & Chattopadhyay, A. Exploring GPCR–Lipid Interactions by Molecular Dynamics Simulations: Excitements, Challenges, and the Way Forward. *J. Phys. Chem. B* **122**, 5727–5737 (2018).

18. Song, W., Yen, H.-Y., Robinson, C. V. & Sansom, M. S. P. State-dependent Lipid Interactions with the A2a Receptor Revealed by MD Simulations Using In Vivo-Mimetic Membranes. *Structure* **27**, 392-403.e3 (2019).

19. Mondal, S., Khelashvili, G., Johner, N. & Weinstein, H. How the Dynamic Properties and Functional Mechanisms of GPCRs Are Modulated by Their Coupling to the Membrane

Environment. in *G Protein-Coupled Receptors* **-** *Modeling and Simulation* (ed. Filizola M.) 55–74 (Springer, Dordrecht, 2014).

20. Liu, W. *et al.* Structural basis for allosteric regulation of GPCRs by sodium ions. *Science* **337**, 232–236 (2012).
21. Anandakrishnan, R., Aguilar, B. & Onufriev, A. V. H++ 3.0: automating pK prediction and the preparation of biomolecular structures for atomistic molecular modeling and simulations. *Nucleic Acids Res.* **40**, W537–W541 (2012).
22. Masureel, M. *et al.* Structural insights into binding specificity, efficacy and bias of a β 2 AR partial agonist. *Nat. Chem. Biol.* **14**, 1059–1066 (2018).
23. Eswar, N. *et al.* Comparative Protein Structure Modeling Using MODELLER. *Curr. Protoc. Protein Sci.* **50**, 2.9.1-2.9.31 (2007).
24. Rasmussen, S. G. F. *et al.* Crystal structure of the β 2 adrenergic receptor-Gs protein complex. *Nature* **477**, 549–557 (2011).
25. Buchan, D. W. A. & Jones, D. T. The PSIPRED Protein Analysis Workbench: 20 years on. *Nucleic Acids Res.* **47**, W402–W407 (2019).
26. Heffernan, R. *et al.* Single-sequence-based prediction of protein secondary structures and solvent accessibility by deep whole-sequence learning. *J. Comput. Chem.* **39**, 2210–2216 (2018).
27. Ring, A. M. *et al.* Adrenaline-activated structure of β 2-adrenoceptor stabilized by an engineered nanobody. *Nature* **502**, 575–579 (2013).
28. Ballesteros, J. A. & Weinstein, H. [19] Integrated methods for the construction of three-dimensional models and computational probing of structure-function relations in G protein-coupled receptors. *Methods Neurosci.* **25**, 366–428 (1995).

29. Dror, R. O. *et al.* Pathway and mechanism of drug binding to G-protein-coupled receptors. *Proc. Natl. Acad. Sci. U. S. A.* **108**, 13118–23 (2011).

30. Mi, L.-Z. *et al.* Structural Basis for Bile Acid Binding and Activation of the Nuclear Receptor FXR. *Mol. Cell* **11**, 1093–1100 (2003).

31. Festa, C. *et al.* Targeting Bile Acid Receptors: Discovery of a Potent and Selective Farnesoid X Receptor Agonist as a New Lead in the Pharmacological Approach to Liver Diseases. *Front. Pharmacol.* **8**, 162 (2017).

32. Di Leva, F. S. *et al.* Binding mechanism of the farnesoid X receptor marine antagonist suvanine reveals a strategy to forestall drug modulation on nuclear receptors. Design, synthesis, and biological evaluation of novel ligands. *J. Med. Chem.* **56**, 4701–4717 (2013).

33. D'Amore, C. *et al.* Design, synthesis, and biological evaluation of potent dual agonists of nuclear and membrane bile acid receptors. *J. Med. Chem.* **57**, 937–954 (2014).

34. Alessandri, R. *et al.* Pitfalls of the Martini Model. *J. Chem. Theory Comput.* **15**, 5448–5460 (2019).

35. Lindorff-Larsen, K. *et al.* Improved side-chain torsion potentials for the Amber ff99SB protein force field. *Proteins Struct. Funct. Bioinforma.* **78**, NA-NA (2010).

36. Cowan-Jacob, S. W. *et al.* The crystal structure of a c-Src complex in an active conformation suggests possible steps in c-Src activation. *Structure* **13**, 861–871 (2005).

37. Luo, Q., Boczek, E. E., Wang, Q., Buchner, J. & Kaila, V. R. I. Hsp90 dependence of a kinase is determined by its conformational landscape. *Sci. Rep.* **7**, 1–11 (2017).

38. Maier, J. A. *et al.* ff14SB: Improving the Accuracy of Protein Side Chain and Backbone Parameters from ff99SB. *J. Chem. Theory Comput.* **11**, 3696–3713 (2015).

39. Midelfort, K. S. *et al.* Redesigning and characterizing the substrate specificity and activity

of *Vibrio fluvialis* aminotransferase for the synthesis of imagabalin. *Protein Eng. Des. Sel.* **26**, 25–33 (2013).

40. Verdonck, S. *et al.* Synthesis and Structure-Activity Relationships of 3,5-Disubstituted-pyrrolo[2,3- b]pyridines as Inhibitors of Adaptor-Associated Kinase 1 with Antiviral Activity. *J. Med. Chem.* **62**, 5810–5831 (2019).

41. Sorrell, F. J., Szklarz, M., Abdul Azeez, K. R., Elkins, J. M. & Knapp, S. Family-wide Structural Analysis of Human Numb-Associated Protein Kinases. *Structure* **24**, 401–411 (2016).

42. Roberts, E., Eargle, J., Wright, D. & Luthey-Schulten, Z. MultiSeq: Unifying sequence and structure data for evolutionary analysis. *BMC Bioinformatics* **7**, 1–11 (2006).

43. Alessandri, R. Multiscale modeling of organic materials: from the Morphology Up. *Dissertation, University of Groningen* (University of Groningen, 2019). doi:10.33612/diss.98150035

44. Melo, M. N., Ingólfsson, H. I. & Marrink, S. J. Parameters for Martini sterols and hopanoids based on a virtual-site description. *J. Chem. Phys.* **143**, 243152 (2015).

45. Jorgensen, W. L. & Tirado-Rives, J. Potential energy functions for atomic-level simulations of water and organic and biomolecular systems. *Proc. Natl. Acad. Sci. U. S. A.* **102**, 6665–6670 (2005).

46. Dodda, L. S., Vilseck, J. Z., Tirado-Rives, J. & Jorgensen, W. L. 1.14*CM1A-LBCC: Localized Bond-Charge Corrected CM1A Charges for Condensed-Phase Simulations. *J. Phys. Chem. B* **121**, 3864–3870 (2017).

47. Dodda, L. S., Vaca, I. C. de, Tirado-Rives, J. & Jorgensen, W. L. LigParGen web server: an automatic OPLS-AA parameter generator for organic ligands. *Nucleic Acids Res.* **45**,

W331–W336 (2017).

48. Wang, J., Wolf, R. M., Caldwell, J. W., Kollman, P. A. & Case, D. A. Development and testing of a general amber force field. *J. Comput. Chem.* **25**, 1157–1174 (2004).

49. Shirts, M. R. & Chodera, J. D. Statistically optimal analysis of samples from multiple equilibrium states. *J. Chem. Phys.* **129**, 124105 (2008).

50. Humphrey, W., Dalke, A. & Schulten, K. VMD: Visual molecular dynamics. *J. Mol. Graph.* **14**, 33–38 (1996).

51. Ishchenko, A. *et al.* Toward G protein-coupled receptor structure-based drug design using X-ray lasers. *IUCrJ* **6**, 1106–1119 (2019).

52. Bonomi, M. *et al.* Promoting transparency and reproducibility in enhanced molecular simulations. *Nature Methods* **16**, 670–673 (2019).

53. Getlik, M. *et al.* Hybrid compound design to overcome the gatekeeper T338M mutation in cSrc. *J. Med. Chem.* **52**, 3915–3926 (2009).

54. Konagurthu, A. S., Whisstock, J. C., Stuckey, P. J. & Lesk, A. M. MUSTANG: A multiple structural alignment algorithm. *Proteins Struct. Funct. Genet.* **64**, 559–574 (2006).

55. Shan, Y. *et al.* How Does a Drug Molecule Find Its Target Binding Site? *J. Am. Chem. Soc.* **133**, 9181–9183 (2011).

56. Kobilka, B. K. Structural insights into adrenergic receptor function and pharmacology. *Trends Pharmacol. Sci.* **32**, 213–218 (2011).

57. Venkatakrishnan, A. J. *et al.* Molecular signatures of G-protein-coupled receptors. *Nature* **494**, 185–194 (2013).

58. Genz, M. *et al.* Alteration of the Donor/Acceptor Spectrum of the (S)-Amine Transaminase from *Vibrio fluvialis*. *Int. J. Mol. Sci.* **16**, 26953–26963 (2015).

59. Genz, M. *et al.* Engineering the Amine Transaminase from *Vibrio fluvialis* towards Branched-Chain Substrates. *ChemCatChem* **8**, 3199–3202 (2016).

60. Nobili, A. *et al.* Engineering the Active Site of the Amine Transaminase from *Vibrio fluvialis* for the Asymmetric Synthesis of Aryl-Alkyl Amines and Amino Alcohols. *ChemCatChem* **7**, 757–760 (2015).

61. Cho, B.-K. *et al.* Redesigning the substrate specificity of ω-aminotransferase for the kinetic resolution of aliphatic chiral amines. *Biotechnol. Bioeng.* **99**, 275–284 (2008).

62. Fersht, A. *Enzyme structure and mechanism*. (Freeman, 1985).

63. Fasella, P. & Hammes, G. G. A Temperature Jump Study of Aspartate Aminotransferase. A Reinvestigation. *Biochemistry* **6**, 1798–1804 (1967).

64. Jardetzky, T. S. & Seville, M. Calculation of rate and equilibrium constants for a ping-pong mechanism from steady-state data. *Biochemistry* **27**, 6758–6763 (1988).

65. Furumo, N. C. & Kirsch, J. F. Accumulation of the Quinonoid Intermediate in the Reaction Catalyzed by Aspartate Aminotransferase with Cysteine Sulfinic Acid. *Arch. Biochem. Biophys.* **319**, 49–54 (1995).

66. Lee, J. *et al.* CHARMM-GUI Input Generator for NAMD, GROMACS, AMBER, OpenMM, and CHARMM/OpenMM Simulations Using the CHARMM36 Additive Force Field. *J. Chem. Theory Comput.* **12**, 405–413 (2016).

67. Huang, J. *et al.* CHARMM36m: An improved force field for folded and intrinsically disordered proteins. *Nat. Methods* **14**, 71–73 (2016).

68. Hills, E. E., Abraham, M. H., Hersey, A. & Bevan, C. D. Diffusion coefficients in ethanol and in water at 298K: Linear free energy relationships. *Fluid Phase Equilib.* **303**, 45–55 (2011).
