# Supplementary material for: Protein–ligand binding with the coarse-grained Martini model
Source: Nat Commun. 2020 Jul 24;11:3714. doi: 10.1038/s41467-020-17437-5 (PMC7382508; doi:10.1038/s41467-020-17437-5)
Supplement: Supplementary file 3 — Description of Additional Supplementary Information [file 41467_2020_17437_MOESM3_ESM.pdf]

Description of Additional Supplementary Files

File Name: Supplementary Movie 1
Description: A binding event of benzene to T4 lysozyme: The benzene molecule samples the solution around the protein for a long time until it finds the binding site, after visiting a pre-pocket near the cavity (represented here by the cyan transparent surface). All residues around the cavity are hydrophobic (yellow), except by a tyrosine (purple). This polar aromatic residue can compete with benzene and pushes the molecule back to the pre-pocket. However, benzene has a higher affinity for the hydrophobic cavity compared to phenol (the side chain analogue of tyrosine). Therefore, eventually benzene binds again to the pocket and stays until the end of the simulation.

File Name: Supplementary Movie 2
Description: Binding and unbinding of adenosine to A2AR. Conversion from AA to CG structure and vice versa is shown prior to and after binding as to highlight the atomistic binding mode of adenosine. Residues involved in the adenosine binding mode are represented as yellow licorice with white labels. Side and top views of the binding processes are shown as insets.
